# Crude Pectic Oligosaccharide Recovery from Thai Chok Anan Mango Peel Using Pectinolytic Enzyme Hydrolysis

**DOI:** 10.3390/foods10030627

**Published:** 2021-03-16

**Authors:** Malaiporn Wongkaew, Bow Tinpovong, Korawan Sringarm, Noppol Leksawasdi, Kittisak Jantanasakulwong, Pornchai Rachtanapun, Prasert Hanmoungjai, Sarana Rose Sommano

**Affiliations:** 1 Interdisciplinary Program in Biotechnology, Graduate School, Chiang Mai University, Chiang Mai 50200, Thailand; malaiporn_wongkaew@cmu.ac.th; 2 Program of Food Production and Innovation, Faculty of Integrated Science and Technology, Rajamangala University of Technology Lanna, Chiang Mai 50300, Thailand; bowtinpovong@rmutl.ac.th; 3 Plant Bioactive Compound Laboratory, Faculty of Agriculture, Chiang Mai University, Chiang Mai 50200, Thailand; 4 Department of Animal and Aquatic Science, Faculty of Agriculture, Chiang Mai University, Chiang Mai 50200, Thailand; korawan.s@cmu.ac.th; 5 Cluster of Agro Bio-Circular-Green Industry (Agro BCG), Chiang Mai University, Chiang Mai 50200, Thailand; noppol@hotmail.com (N.L.); jantanasakulwong.k@gmail.com (K.J.); pornchai.r@cmu.ac.th (P.R.); 6 School of Agro-Industry, Faculty of Agro-Industry, Chiang Mai University, Chiang Mai 50200, Thailand; prasert.h@cmu.ac.th

**Keywords:** *Bifidobacterium animalis*, fruit peel pectin, *Lactobacillus reuteri*, molecular weight, pectinase, prebiotic activity, short chain fatty acid, waste valorisation

## Abstract

Pectin recovered from mango peel biomass can be used as a potential source for pectic oligosaccharide hydrolysate with excellent probiotic growth-enhancing performance and prebiotic potentials. Consequently, the objectives of the current study were to optimise the enzyme hydrolysis treatment of mango peel pectin (MPP) and to evaluate the pectic oligosaccharide effects of *Lactobacillus reuteri* DSM 17938 and *Bifidobacterium animalis* TISTR 2195. Mango of “chok anan” variety was chosen due to its excessive volume of biomass in processing and high pectin content. The optimal treatment for mango peel pectic oligosaccharide (MPOS) valorisation was 24 h of fermentation with 0.3% (*v/v*) pectinase. This condition provided small oligosaccharides with the molecular weight of 643 Da that demonstrated the highest score of prebiotic activity for both of *B. animalis* TISTR 2195 (7.76) and *L. reuteri* DSM 17938 (6.87). The major sugar compositions of the oligosaccharide were fructose (24.41% (*w/w*)) and glucose (19.52% (*w/w*)). For the simulation of prebiotic fermentation, *B. animalis* TISTR 2195 showed higher proliferation in 4% (*w/v*) of MPOS supplemented (8.92 log CFU/mL) than that of *L. reuteri* (8.53 CFU/mL) at 72 h of the fermentation time. The main short chain fatty acids (SCFAs) derived from MPOS were acetic acid and propionic acid. The highest value of total SCFA was achieved from the 4% (*w/v*) MPOS supplementation for both of *B. animalis* (68.57 mM) and *L. reuteri* (69.15 mM). The result of this study therefore conclusively advises that MPOS is a novel pectic oligosaccharide resource providing the opportunity for the sustainable development approach through utilising by-products from the fruit industry.

## 1. Introduction

Pectin is mostly required in the food industry owing to its additive ability to form food hydrogels or emulsions, which alter texture and firmness of food products [1]. The soluble properties with structural complexity warrant its importance as a functional ingredient with various health benefits claimed [2,3]. Structurally, pectin is a complex hetero-polysaccharide mainly comprised of α-1,4-D galacturonic acids (≈70%) known as homogalacturonan [4,5,6,7,8]. Their structures are also comprised of various monosaccharides, particularly those of glucose, mannose, galactose and arabinose [4,5,9]. As a consequence, pectin is also known as a source of oligomers with the prebiotic potential that is currently in high demand in food and pharmaceutical industries [10]. However, prior to application, pectin must be hydrolysed to short chain oligosaccharides for better enhancement of probiotic growth performance and formation of fermented by-products [11].

Prebiotics are identified as non-digestible food constituents that benefit hosts by selectively enhancing the growth of probiotic bacteria (mainly the genus of Bifidobacterium and Lactobacillus) and reducing pathogenic effects of harmful bacteria by producing short chain fatty acids (SCFAs; mainly acetic, propionic and butyric acids) [12,13]. *B. animalis* and *L. reuteri* are especially well-studied probiotic strains that can be found in different parts of the human body and are able to withstand a low pH in the stomach and contact with bile in the small intestine [14,15]. Consequently, microbiota balance can promote human health by stimulating the immune system, synthesising vitamins and improving digestion and absorption of essential nutrients [16,17,18,19]. Pectic-oligosaccharide (POS) is a prebiotic that has recently gained attention as a novel functional food ingredient [20,21,22,23,24,25,26]. POS is generally obtained from partial depolymerisation of pectin-rich agro-residues through enzymatic hydrolysis [9,10,27,28,29]. The enzymes digest pectin to monosaccharides or oligosaccharides through regio- and stereoselectivity [30,31]. By this technique, the obtained oligosaccharides are mostly composed of carbon sources for probiotics depending on the types of raw materials [6].

Several studies have investigated POS recovery from different fruit biomass using enzymatic hydrolysis, such as from citrus peel [9], lemon peel [32], sugar beet pulp [32] hawthorn [33] and orange peel [28]. Citrus peel is an important source of intermediate pectin for POS recovery. Gomez et al. [34] revealed that recovering POS with commercial pectinase from the hydrolysate of lemon peel waste illustrated high content of arabinose and galactose and other oligosaccharides. The POS of this type could complement prebiotic growths, and are thereby candidates that exert a number of health-promoting effects. Ho et al. [9] and Zhang et al. [10] found that POS derived from citrus pectin could enhance growth, fermented products and acid tolerance of probiotics. Thailand is one of the major fruit producing and processing countries where biomass is generated enormously and attempts have been made to value add these by-products through valorisation as to comply with the government policies on zero-waste production [35,36,37]. However, none of those attempts have investigated pectic oligosaccharide components from these resources.

Approximately 300,000 tons of ripe mangoes (*Mangifera indica* L.) are used in Thailand for processing, mainly in the puree, frozen fresh cut, drying and canning industries with the preferred cultivars being “kaew”, “chok anan”, “mahachanok” and “nam dok mai” [38]. As a result, fairly high amounts of mango by-products (peel, pulp and kernel) are generated which largely have adverse impacts on the environment [39]. These by-products account for 35–60% of the total fruit weight [40] and the cost of elimination of such biological mass is not only costly but also generates a large carbon footprint. Therefore, they are mainly fed to animals or disposed of in the environment [41]. Biomass mango peel accounts for 20% of the total fruit weight, therefore is a potential source of dietary fibre with high recovery of pectin (5–10%) depending on the extraction methods and fruit varieties [42,43,44,45,46]. A previous study revealed that peel from the Thai mango variety “chok anan” provided a substantially high amount of pectins (13%), mainly of low methoxyl level with elevated gelation properties at low sugar content, and thus it has been widely used as an additive in dietary food and beverages [47]. Nonetheless, as mentioned, POS recovery and its characteristics from Thai mango peel have not been thoroughly explored. With this rationale, the objectives of this research were first to optimise the hydrolysis condition of mango peel pectin and then to evaluate the advantages of POS on the fermentation by *L. reuteri* and *B. animalis*. The outcome of this study not only provides an alternative way to add value to by-products from Thai fruit industry but also affords a feasible industrial model towards sustainable development.

## 2. Materials and Methods

### 2.1. Chemicals

Standard free fatty acids (acetic acid, propionic acid, isobutyric acid, butyric acid, isovaleric acid, valeric acid) were purchased from Restex Corporation (Bellefonte, PA, USA). Monosaccharide standards, namely D-glucose, D-fructose, D-xylose, D-galactose and L-arabinose were supplied by Loba (Loba Chemie Pvt Ltd., Mumbai, India), Sigma-Aldrich (St. Louis, MO, USA) and Ajax Finechem (Ajax Finechem Pty Ltd., Sydney, Australia). L(+)-Lactic acid standard was purchased from Sigma-Aldrich (St. Louis, MO, USA). Chemicals for pectin extraction and analysis of indigestible polysaccharide were supplied by Sigma-Aldrich (St. Louis, MO, USA), RCI Labscan Limited (Bangkok, Thailand) and AppliChem GmbH-An ITW Companies (Darmstadt, Germany). Dextran was used as an oligosaccharide standard (Sigma-Aldrich, St. Louis, MO, USA). Bacterial supplemented media, including de Man-Rogosa-Sharp broth (MRS) and Luria-Bertani [48] broth, were ordered from Becton, Dickinson and Company (Spark, MD, USA).

### 2.2. Microorganisms

Two probiotic bacterial strains were used in this experiment. *L. reuteri* DSM 17938 (Protectis^®^) was a commercial probiotic (BioGAia^®^ Drops, made in Sweden). *B. animalis* 2195 was obtained from Thailand Institute of Scientific and Technological Research (TISTR, Bangkok, Thailand). The inoculate of lactobacilli was cultured in the MRS broth [49]. *Bifidobacterium* was also cultivated in MRS broth supplemented with bacto soytone (5.0 g/L) [50]. *Escherichia coli* 117 was used as enteric bacteria which was acquired from the TISTR and sub-cultured in an appropriate medium of Luria-Bertani broth [48,51]. All microorganisms were cultivated under anaerobic conditions in a CO_2_ incubator (Thermo Fisher Scientific Inc., Waltham, MA, USA).

### 2.3. Optimisation Condition of Pectic Oligosaccharide Preparation from Mango Peel

Mango peel was removed from fully ripe “chok anan” mangoes (L = 69.98 ± 2.72, a* = 5.55 ± 0.73, b* = 43.09 ± 6.68; peel thickness = 1.69 ± 0.14 mm; percentage of peel to fruit weight = 14.39 ± 0.57%; ρ = 1.247 ± 0.07 g/cm^3^). Pectin was extracted from the dried peel powder using a microwave oven (ME711K-XST, Samsung, Bangkok, Thailand) at 700 watt-power for 3 min using acidic solution (pH 1.5) that yielded ca.15% (*w/w*) [45,52]. The chemical characteristics of pectin extracted from “chok anan” peel are illustrated in Table S1. The filtrate was centrifuged at 5000× *g* for 20 min and then pectin was precipitated from the supernatant using the same volumes of ethanol (95%). The separation was achieved by vacuum filtration. The obtained mango peel pectin (MPP) was dried in a hot air-oven at 40 °C until constant weight [53].

In addition, the previous extracted MPP was treated with a commercial pectinase enzyme (Pectinex^®^ ultra tropical, Novozymes Malaysia Sdn Bhd, Kuala Lumpur, Malaysia) at 6, 12 and 24 h of hydrolysis intervals and at the concentrations of 0.1, 0.2 and 0.3% (*v/v*) [54]. Prior to the experiment, the activity of Pectinex was checked (20 Unit/mL). The MPP was prepared to 2% pectin solution in 0.02 M acetate buffer (pH 4.5). After hydrolysis, all samples were heated in a boiling water for 10 min to deactivate pectinase activity. After cooling down to room temperature, it was then centrifuged at 5000× *g*, 15 min. The supernatant was collected and dehydrated using a vacuum dryer at 50 °C until the moisture content was 7% [9]. Each sample was examined for quality assessments as following.

### 2.4. MPOS (Mango Peel Pectic Oligosaccharide) Quality Assessments

#### 2.4.1. Determination of Molecular Weight

The molecular weights (M_w_) of MPOS (mango peel pectic oligosaccharide) were determined by high-performance size-exclusion chromatography method (gel permeation) according to a modified technique of Yang et al. [55] and Ho et al. [9]. A sample solution (20 μL) was injected into a Ultrahydrogel Linear 1 Column (Waters 600E, Milford, MA, USA) using the mobile phase comprising of 0.8 M sodium chloride at the flow rate of 0.6 mL/min and column temperature was 30 °C. The M_w_ of MPOS was determined by comparing the sample retention time with the standard curve of dextran standard series (4.0–401.0 kDa).

#### 2.4.2. Determination of Monosaccharide Compositions

Qualitative and quantitative analyses of the monosaccharides in MPOS samples were performed using high performance liquid chromatography (HPLC) according to the modified methods of Tieking et al. [56] and Schwab and Ganzle [57]. The sample solutions were diluted three times, then filtered through a 0.22 nylon filter and 10 µL diluted samples were determined for types and contents of monosaccharides. The HPLC used was Shimadzu RID-20A Chromatopac, Japan, with column Agilent Zorbax LC-NH_2_, 4.6 mm × 250 mm, 5 µm. The mobile phase was acetonitrile:water at 75:25, 1.0 mL/min flow rate at ambient temperature and refractive index (RI) detector. Five monosaccharides (arabinose, xylose, glucose, galactose and fructose) were chosen as the standards. All analyses were done in triplicates.

#### 2.4.3. Selection of MPOS Condition Using Prebiotic Activity

Prebiotic activity analysis was determined using a bacteria count technique according to Zhang et al. [10]. Briefly, 1% (*v/v*) of a twice-activated culture of *L. reuteri* DSM 17938 and *B. animalis* TISTR 2195 was added to both of the MRS media containing 2% (*w/v*) glucose and 2% (*w/v*) MPOS samples. The cultures were incubated at 37 °C for 48 h under anaerobic system in the CO_2_ incubator. At 0 and 48 h of the fermentation process, inoculated samples were numbered in triplicates using the serial dilution method on MRS agar and the results were calculated as CFU/mL of culture [10]. The quantitative score of prebiotic activity reported by Huebner et al. [58] can be calculated according to the following equation (Equation (1)):

(1) $$\begin{array}{ll} \mathrm{Prebiotic}\;\mathrm{activity}\; & \mathrm{score}\;=\;\left[\frac{(\mathrm{probioticlogCFU}/\mathrm{mL}\;\mathrm{on}\;\mathrm{the}\;\mathrm{prebiotic}\;\mathrm{at}\;48\;h\;-\;\mathrm{probioticlogCFU}/\mathrm{mL}\;\mathrm{on}\;\mathrm{the}\;\mathrm{prebiotic}\;\mathrm{at}\;0\;h}{(\mathrm{probioticlogCFU}/\mathrm{mL}\;\mathrm{on}\;\mathrm{glucose}\;\mathrm{at}\;48\;h\;-\;\mathrm{probioticlogCFU}/\mathrm{mL}\;\mathrm{on}\;\mathrm{glucose}\;\mathrm{at}\;0\;h}\right]\\ & -\;\left[\frac{(\mathrm{enteric}\;\mathrm{logCFU}/\mathrm{mL}\;\mathrm{on}\;\mathrm{the}\;\mathrm{prebiotic}\;\mathrm{at}\;48\;h-\;\mathrm{entericlogCFU}/\mathrm{mL}\;\mathrm{on}\;\mathrm{the}\;\mathrm{prebiotic}\;\mathrm{at}\;0\;h}{(\mathrm{entericlogCFU}/\mathrm{mL}\;\mathrm{on}\;\mathrm{glucose}\;\mathrm{at}\;48\;h\;-\;\mathrm{entericlogCFU}/\mathrm{mL}\;\mathrm{on}\;\mathrm{glucose}\;\mathrm{at}\;0\;h}\right] \end{array}$$

A higher score demonstrates a higher prebiotic activity [10]. The MPOS treatment representing the highest score of prebiotic activity was then selected for the simulation of the probiotic fermentation.

### 2.5. Fermentation of MPOS on Probiotic Growth and Products

Glucose-free MRS and *Bifidobacterium* broths were used as the base media for *L. reuteri* DSM 17938 and *B. animalis* TISTR 2195, respectively. Both media were supplemented with 1%, 2% and 4% (*w/v*) of MPOS obtained from the selected treatment. Each medium was then inoculated with 10^4^ CFU/mL of the probiotic cultures. The glucose-free broth (negative control) with the supplementation of 2% glucose was applied as the positive control. After incubation at 37 °C in a 20 mL test tube for 0, 24, 48 and 72 h under anaerobic condition in the CO_2_ incubator, the media were determined for indigestible oligosaccharide, probiotic population, acidity alteration and short chain fatty acid production.

#### 2.5.1. Indigestible Oligosaccharide

The content of oligosaccharide in the fermented samples was evaluated using the modified method of indigestible polysaccharides (oligosaccharides) after Wichienchot et al. [59]. All MPOS samples were analysed for reducing sugar contents using the modified dinitrosalicylic acid method [60] and total sugar contents with the modified phenol sulfuric method [61]. MPP was used as a control. The indigestible oligosaccharide content (mg/g dry MPOS) in the samples was calculated from (Equation (2)):

Indigestible oligosaccharide (mg/g) = Total sugar after digestions (mg/g) − Reducing sugar before the digestions (mg/g) (2)

#### 2.5.2. Simulation of the Fermentation

##### Probiotics Population

The population of probiotic bacteria in the cultivation media was evaluated by the optical density of all samples using C30M portable spectrophotometer (PG Instruments Limited, UK) at 600 nm (OD_600_). The cell number corresponding to the OD_600_ reading was calculated from a calibration curve of *L. reuteri* DSM 17938 and *B. animalis* TISTR 2195 and on average 1.0 OD_600_ unit corresponded to 4.0 × 10^8^ and 7.0 × 10^9^ CFU/mL, respectively. The calibration curves of both cultures were generated by cultivating the bacteria until their OD_600_ reached 1.0. Cultures were then diluted to four or five different concentrations and enumerated on MRS agar at 37 °C for 24–48 h under anaerobic conditions. The calibration curves were generated by plotting the bacterial concentrations (CFU/mL) versus OD_600_ [62].

##### pH Value

The level of pH was analysed as fermentation indicators. The pH value was measured directly in the media samples by the pH meter (Mettler-Toledo, Greifensee, Switzerland).

#### 2.5.3. By-Products of Probiotics

##### Lactic Acid

Lactic acid content (LA) was determined by HPLC techniques using Shimadzu LC-20AD (Shimadzu Corporation, Kyoto, Japan) equipped with a low pressure quaternary gradient pump along with the dual wavelength UV-Visible detector, column oven and auto sampler after the modified method of Kishore et al. [63]. The column oven temperature was maintained at 25 °C and the chromatographic separation was attained using Ultra Aqueous C18 column (250 mm × 4.6 mm ID, 5 µm) (Restex Corporation, Bellefonte, PA, USA). The isocratic elution was achieved with 50 mM potassium phosphate (pH 2.5) as mobile phase. The flow rate was maintained at 1.0 mL/min and the injection volume was 10 µL. The effluent was observed at a wavelength of 210 nm. The LA calibration standards were prepared by serial dilutions (0.3–1.2 mg/mL) in 50 mM potassium phosphate (pH 2.5).

##### Short Chain Fatty Acid Production

The supernatants from the anaerobic culture inoculated with probiotic cultures were analysed for short chain fatty acid (SCFA), including acetic acid, propionic acid, isobutyric acid, butyric acid, isovaleric acid and valeric acid using gas chromatography, Nexis GC-2030, Shimadzu according to the modified method of Filipek and Dvorak [64]. A Rtx^®^-1 capillary column (nonpolar phase) Crossbond dimethyl polysiloxane was used, 15 m × 0.53 mm ID × 5 μm (Restek, Bellefonte, PA, USA). Carrier gas (helium) at flow rate 3.0 mL/min, detector-FID, temperature program used: 60–200 °C (20 °C/min, 10 min), injector: 250 °C, detector: 300 °C. The injector was equipped with a glass liner of glass wool to separate particles of dirt from the sample. The samples were dosed by an AOC-20i Plus (Shimadzu corporation, Kyoto, Japan) automatic dosing device at an injection size of 1.0 μL using the split method and a 30:1 splitting ratio. The calibration standards were prepared by serial dilutions to obtain concentrations of 25–1000 μg/mL.

### 2.6. Statistical Analysis

All experiments were done in at least triplicates for each test. For the experimental design, the 3 × 3 factorial completely randomised design (CRD) was used and the data were analysed using two-way analysis of variance (ANOVA) with Duncan’s multiple range test. Difference in values was considered significantly different when the *p* value was < 0.05. All statistical analysis was performed using IBM SPSS program v. 23.0 (Armonk, New York, NY, USA) (Supplementary Materials Tables S3–S8). The relationships between the monosugar compositions and prebiotic activity as well as MPOS concentrations and probiotic growth were analysed using principal component analysis (PCA) by the XLSTAT v. 2020 (Addinsoft, New York, NY, USA).

## 3. Results and Discussion

### 3.1. Optimisation Condition of MPOS on Probiotic Growth

#### 3.1.1. Monosaccharide Contents and Molecular Weight of MPOS s

Molecular weights (M_w_) of MPOS obtained from MPP hydrolysed with various treatments are shown in Table 1. The M_w_ of all MPOS was less than 1000 Da. We noticed that the longer the hydrolysis time and pectinase concentrations, the lower the M_w_ obtained as described as M_z_ values. This finding was in line with other studies [65,66]. The smaller M_w_ oligosaccharides produced during the hydrolysis depend upon the greater proportion of monosaccharides [9]. Monosaccharide compositions of MPOS s are illustrated in Table 1. Among the analysed sugars, fructose and glucose were the most abundant monosaccharides in all MPOS samples, followed by galactose and arabinose and the levels increased significantly with any hydrolysis time (*p* < 0.05). We also found that the higher the amounts of enzyme, the better yields of each monosaccharides obtained. At 24 h of hydrolysis time, the maximum yield of glucose (19.0%), fructose (24.0%), galactose (3.3%) and arabinose (3.0%) were achieved. Alteration of sugar types and their concentration compositions may vary upon the source of raw materials, hydrolysis time and pectinase concentration [9,24,34]. Ivanova et al. [65] and Grahame et al. [66] added that the increased amounts of sugars followed zero-order reaction between substrate and enzyme. The highest monosaccharide concentration was with the treatment of 0.3% pectinase after 24 h hydrolysis and the lowest was with 0.1% pectinase after hydrolysed for 6 h in all sugar types. Furthermore, Cano et al. [67] and Dasaesamoh et al. [68] described that the pectinase practically cleaves ester and glycosidic bonds, thereby releasing the oligosaccharides and monosaccharides.

#### 3.1.2. Prebiotic Assessment

**Figure 1:**
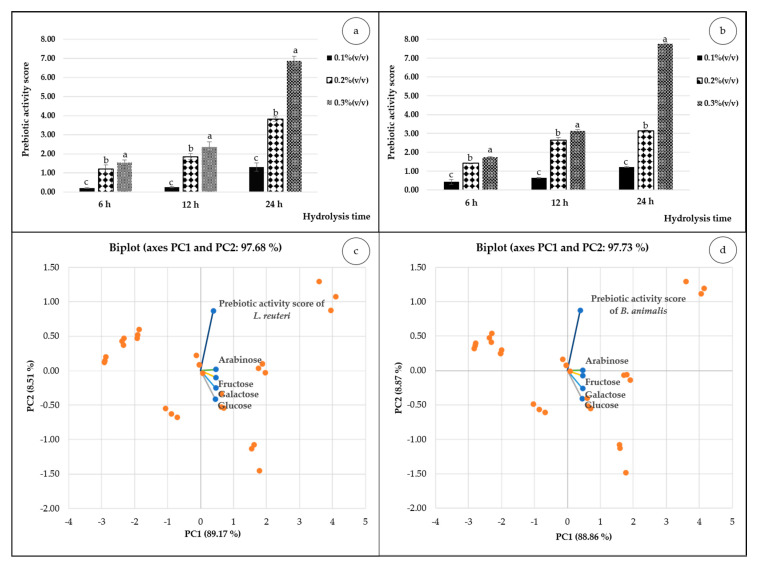
Prebiotic activity scores of MPOS obtained from MPP hydrolysed using various concentrations of pectinase (0.1, 0.2, 0.3% (*v/v*)) at different hydrolysis times (6, 12, 24 h) of *L. reuteri* DSM 17938 (**a**) and *B. animalis* TISTR 2195 (**b**). Different letters on the bars in the same hydrolysis times indicate statistically significant differences (*p* < 0.05). The chemometric PCA of monosaccharide compositions and prebiotic activity scores of *L. reuteri* DSM 17938 (**c**) and *B. animalis* TISTR 2195 (**d**).

Prebiotic activity scores of MPOS for *L. reuteri* and *B. animalis* are illustrated in Figure 1. The maximum scores for *L. reuteri* (6.87) and *B. animalis* (7.76) were obtained from the same hydrolysis condition of 0.3% (*v/v*) pectinase at 24 h interval time. On the contrary, the lowest scores of both probiotics were from 0.1% (*v/v*) enzyme concentration at 6 h. The higher prebiotic score indicates the greater growth performance of probiotics according to Huebner et al. [58]. Results in Figure 1 also showed the positive correspondence of pectinase concentrations and incubation time. Similar results were also reported by Thitiratsakul and Anprung [69] as well as Fasawang and Anprung [70]. Ho et al. [9] also revealed that POS preparation from citrus pectin using greater content of pectinase and longer hydrolysis time provided lower average molecular weights (monosaccharides and small oligosaccharides). Presumably, great amount of pectinase and longer time of degradation cleaved pectin to be molecules influencing the better prebiotic effectiveness [6]. In addition, the hydrolysis of pectin also enhances the release of bound bioactive compounds, and these affect higher growth of the probiotic bacterial strains (*L. acidophilus* La5) as well as greater score of prebiotic [69]. As a result of this study we also found that the higher the content of pectinase and longer hydrolysis time, the lower the molecular weights of MPOS obtained (Table 1).

By comparison, the scores of both probiotics showed that *B. animalis* gave remarkably higher scores than *L. reuteri* in all hydrolysis conditions. Likewise, the same probiotic genus of *B. bifidum* also provided significantly higher scores than those of *L. paracasei* when using POS from citrus peel pectin [10]. This is also in agreement with results reported by Gopal et al. [71]. Therefore, *Bifidobacterium* could hydrolyse the oligosaccharide source due to its specific enzyme (β-galactosidase), which was responsible for their growth using galactan as a substrate vastly available in plant-based prebiotics [72,73]. To have a look at the influence of monosaccharide compositions on prebiotic activity scores of both probiotics, we used PCA. The results showed that the score plots depicted > 97% of PC1 and PC2 in both cases (Figure 1c,d). The biplot analysis indicated that the arabinose had the most influence in growth of probiotics followed by fructose, galactose and glucose. This finding is in line with POS extracted from citrus peel where arabinose was the most usable monosaccharide by *L. paracasei* and *B. bifidum* [10].

### 3.2. Fermentation of MPOS on Probiotic Growth and Products

#### 3.2.1. Indigestible Oligosaccharide

**Figure 2:**
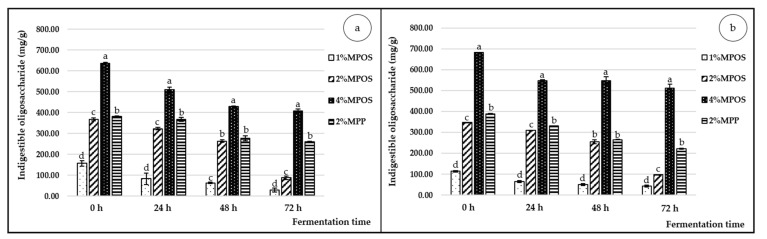
Indigestible oligosaccharide of *L. reuteri* DSM 17938 (**a**) and *B. animalis* TISTR 2195 (**b**) in the media supplemented with different contents of MPOS and 2% MPP (control) cultivated for 72 h of fermentation time. Different letters on the bars in the same fermentation time indicate statistically significant differences (*p* < 0.05).

The amounts of oligosaccharides after fermentation of MPOS using various concentrations are shown in Figure 2. Initially (T_0_), the oligosaccharide contents in the media of *L. reuteri* (Figure 2a) and *B. animalis* (Figure 2b) were in the range of 156.97–635.23 mg/g and 113.68–682.92 mg/g, respectively, which varied depending on the concentrations of added MPOS (1–4% (*w/v*)). Subsequently, oligosaccharide contents in all treatments decreased continuously throughout the fermentation period and the lowest value was at 72 h. The controlled prebiotic treatment (MPP) remained quite stable during the fermentation period. Consequently, the greater the fermentation time, the more the degradation of oligosaccharide occurred because the partial oligosaccharides were digested by the probiotic bacteria and used as a carbon source for their growth and product formation (lactic acid and short chain fatty acids) [9]. Figueroa-Gonzalez et al. [74] found high reduction in galactooligosaccharide or GOS content by *Lactobacillus* strains after fermentation period for 24 h. In addition, Cheng et al. [75] also reported that long maintenance of GOS in mice colon (3 weeks) affected the increased abundance of *Bifidobacterium* because its β-galactosidases could efficiently degrade GOS to a much more utilisable form that enhanced its growth and performance. For the control treatment (MPP), the substrate content decreased lower than all MPOS in both bacterial strains. This incidence may correspond to the degree of esterification of the long chain polysaccharides, giving slower degradation than that of the low-esterified substrate [20,76]. The results of this present study showed that *L. reuteri* and *B. animalis* were capable of utilising the MPOS as a substrate, however the ability varied among the species and substrate contents [74]. Moreover, both strains could use the MPOS better than the MPP, which indicated that the MPOS had more efficiency thereby promoting higher growth of the probiotics than that of MPP.

#### 3.2.2. Probiotics Population

**Figure 3:**
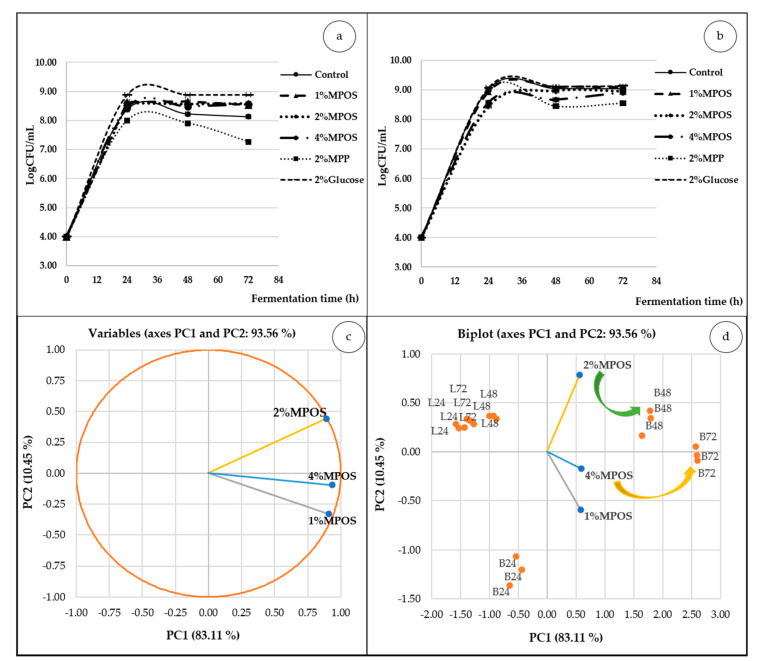
The populations (log CFU/mL) of *L. reuteri* DSM 17938 (**a**) and *B. animalis* TISTR 2195 (**b**) inoculated at 4 log CFU/mL in the medium supplemented with different carbon sources. The chemometric PCA ((**c**) = score plot and (**d**) = biplot) illustrates the relationship between prebiotic concentration and probiotic growth (L = *L. reuteri* and B = *B. animalis*) at each fermentation time (24, 48 and 72 h). Error bars represent standard deviation (*p* < 0.05).

Growths of *L. reuteri* and *B. animalis* over 72 h in various carbon sources at different fermentation times illustrated the same patterns as shown in Figure 3. The number of cells rapidly increased within 24 h, then gradually rose, declined and were maintained until 72 h. Similarly, Kneifel [77] also mentioned the growth characteristics of *Bifidobacterium* strain in several prebiotic sources that continuously increased and statically assessed after 24 h of incubation time. In accordance with a general phase of bacterial growth, the log or exponential phase of microorganisms is within 24 h because the bacteria operate rapid reproduction and cell doubling which occurs every few minutes. Subsequently, the declining and stationary phase appear after 24 h of fermentation time due to the depletion of available nutrients and the accumulation of waste products [78]. Compared to the negative controls, viz. carbon source and MPP, they presented significantly lower growth of both *L. reuteri* and *B. animalis* than those of MPOS. Likewise, crude pectin extracted from sugar beet pulp showed lower response in growth of *Lactobacillus* and *Bifidobacterium* [36]. These results could be explained in that the longer the chain of pectin, the higher the degree of esterification with greatly methylated carbon sources that were more difficult to hydrolyse [20]. In the case of the positive control (glucose), it was found that the increase of cell density of both bacterial strains was significantly higher than that of MPOS at all incubation times. The result was in accordance with Soto [79] and Goderska et al. [80] who reported the higher growth trend of *Lactobacillus* in the MRS supplemented with glucose.

To have a closer look at the relationship of prebiotic concentration and probiotic growth of two probiotic types (*L. reuteri* and *B. animalis*), we then utilised the chemometric PCA. The first two dimensions of the PCA accounted for a total of 93.56% across the PCA score plot (PC1; 83.11% and PC2; 10.45% of the variance) (Figure 3c). It was also apparent that the MPOS-supplemented medium was active only with *B. animalis* (Figure 3d). This may be a result of the intracellular enzymes of *Bifidobacterium* which could hydrolyse the oligosaccharides into monosaccharides (glucose and fructose phosphates) and utilise them as a nutrient source [9,10]. In addition, Olano-Martin et al. [20] found that the oligosaccharide (apple pectin) delivered less growth performance with *Lactobacillus*.

It is worthwhile to note in the same figure that 2% MPOS corresponded well with *B. animalis* at 48 h of incubation time, while the higher concentration (4% MPOS) gave a good response with 72 h of fermentation time. We then assumed that higher viability of *B. animalis* required higher concentration of pectic oligosaccharide. In line with this, Ho et al. [9] reported that *Bifidobacterium* had the highest growth in the media containing 4% POS from citrus pectin, followed by 2% and 1% (*w/v*). They suggested that the higher the oligosaccharide concentration, the more carbon sources for bacterial survivors obtained.

#### 3.2.3. pH and Lactic Acid

**Figure 4:**
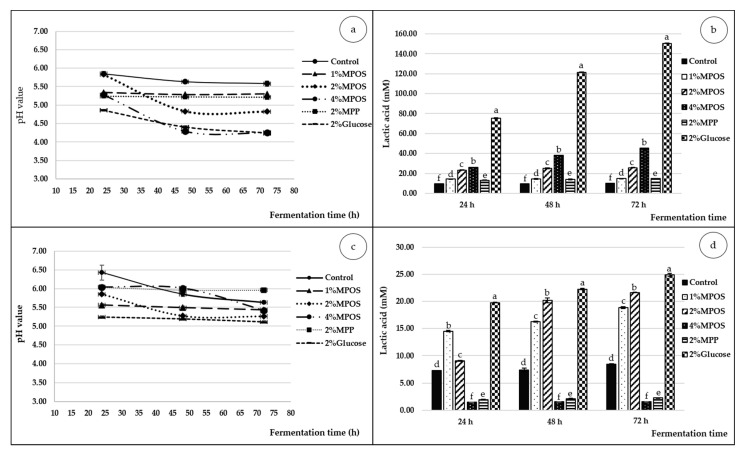
pH and lactic acid of *L. reuteri* DSM 17938 (**a**,**b**) and *B. animalis* TISTR 2195 (**c**,**d**) cultivated in media supplemented with different carbon sources for 72 h of fermentation time. Different letters on the bars in the same fermentation time indicate statistically significant differences (*p* < 0.05).

The acidity alteration described as pH and lactic acid concentrations of media supplemented with various carbon sources is illustrated in Figure 4. Lactic acid is known as a by-product of bacterial anaerobic fermentation and is responsible for the reduction of pH in the media [9,81,82,83,84]. As shown in the figure, low pH and high lactic acid content (LA) were attained in both bacterial strains at longer fermentation time due to the oligosaccharide structure of MPOS substrate that was gradually degraded to small molecules of sugars which were later converted to LA via an anaerobic glycolysis pathway by the probiotic bacteria [85].

Different carbon sources obviously affected types of acidic products. The negative controls illustrated a higher pH and lower LA, while the positive control (glucose) largely provided the contrary results. It could be stated that glucose was metabolised rapidly by the probiotics as a non-prebiotic simple carbon source [86], thus a greater amount of LA and lower value of pH was achieved. This is in agreement with Usta-Gorgun and Yilmaz-Ersan [86] who also reported that glucose gave the lowest pH value, when correlated with media containing the prebiotics from orchid root. For prebiotic pectin, the higher the concentration of MPOS, the lower the pH value and the higher the lactic acid production obtained. This could be also explained by the zero order in substrate concentration and product formation [87]. Moreover, each bacterial strain can alter its fermentation ability to generate the distinctive acidic products when cultivated on different concentrations of oligosaccharides [65].

In comparison with the two tested bacterial strains, *L. reuteri* provided higher acidity and LA production in the media than that of *B. animalis*. These results were the same as reported in previous studies [9,80]. The higher LA content of *L. reuteri* was involved with the ability of enzymatic production (L-lactate dehydrogenase) for converting monosaccharides obtained from oligosaccharide degradation to lactic acid [48].

#### 3.2.4. Short Chain Fatty Acid Production (SCFA)

**Figure 5:**
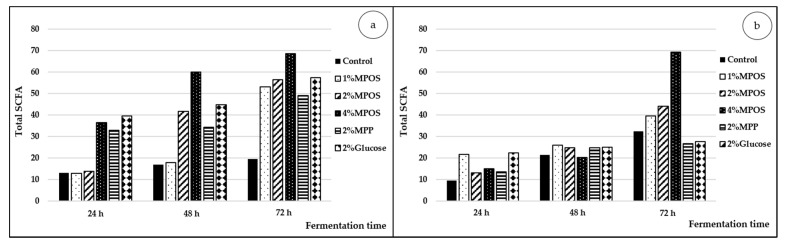
Total short chain fatty acid production of *L. reuteri* DSM 17938 (**a**) and *B. animalis* TISTR 2195 (**b**) cultivated in media supplemented with different carbon sources for 72 h of fermentation time.

SCFAs are generally produced through hexose and pentose pathways from the digestion of fibre and non-digestible carbohydrates of plant natural resources by probiotic bacteria [88,89]. Polysaccharide derived from fruits is known as the main source of SCFAs that promote human health benefits, including the reduction of harmful bacteria such as *Clostridium* and the enhancement of beneficial bacteria, as well as the stimulation of the intestinal immune system [90,91]. Montoya et al. [92] reported that kiwifruit fibre was a good source of SCFAs in both in vivo and in vitro fermentation systems. Changes in total SCFA contents after 24, 48 and 72 h of fermentation supplemented with different carbon sources are shown in Figure 5 (with the amounts of individual compounds shown in Supplementary Materials Tables S1 and S2). SCFAs were detected primarily at 24 h and increased consequently thereafter in all treatments for both of *L. reuteri* (Figure 5a) and *B. animalis* (Figure 5b). The initial total values were between 12.77–39.31 mM for *L. reuteri* and 9.48–22.19 mM for *B. animalis*. Similarly, fermentation time dependency with amount of SCFAs has been seen in other studies [33,34,35,93,94]. It is advised that bacteria usually digest dietary fibres to monosaccharides using glycoside hydrolases and then to SCFAs as the fermented products through carbon metabolic pathways during anaerobic fermentation. Thus, higher contents of end-products can be found much later in the fermentation process [95].

Among samples added with MPOS, the highest total SCFA value was obtained from the 4% supplementation, followed by that of 2% and 1%, whereas the negative CTRLs showed much lower concentrations. It appeared that a higher availability of substrates affected high production of SCFAs. Another factor that influenced SCFA production was the structure of the substrates. For example, oligosaccharide soluble fibres (i.e., fructooligosaccharides) gave a higher amount of SCFAs than the soluble fibres (i.e., longer-chain pectin), which may be due to the complex structure of pectin that limits the accessibility of bacteria and hydrolytic enzymes [96,97]. Gulfi et al. [98] added that the fermentation rate of partially hydrolysed pectins depended largely on their complexed structures.

The main SCFAs produced by both *L. reuteri* and *B. animalis* were acetic acid and propionic acid (as shown in the Supplementary Materials Tables S1 and S2). Both acids are known as the main SCFAs derived from pectic oligosaccharide fermentation [76]. Gómez et al. [36] also found that acetic acid was the most abundant SCFA, followed by propionic acid and butyric acid in pectic oligosaccharide obtained from lemon peel waste and sugar beet pulp. This is related to the dynamics of microbial population; these resources promote the growth of *Bifidobacteria* and *Lactobacilli* as acetate producers.

To further describe the qualitative and quantitative assessments of each carbon sources, we then presented the production ratio between LA and the total SCFAs as in Table 2. When compared only with the MPOS-supplemented samples, the highest ratios of ΔLA/ΔTotalSCFA for *L. reuteri* and *B. animalis* were 4% and 2%, respectively, which was clearly associated with the lactic acid content (Figure 2). It could be elucidated that the microbiota mostly generates LA as a common short chain hydroxy-fatty acid in intestinal lumen, in which it can be diverted to other SCFAs by lactate-fermenting bacterial species [99,100]. Similar findings also revealed that the supplementation of 2% orchid root fibre in the cultivation media of *B. bifidum* showed the highest ratio of ΔLA/ΔTotalSCFA [86]. In the case of the positive control, the maximum ratio of ΔLA/ΔTotalSCFA was simply recognised because the small molecule was easily transformed to a substantial amount of LA and SCFAs by both bacterial strains, whereas the negative controls had relatively low ratio values, which were also related to the production ability of LA and total SCFA.

## 4. Conclusions

In order to hydrolyse mango peel pectin to a pectic oligosaccharide form (MPOS), longer incubation and higher pectinase concentration were suggested. The monosaccharide compositions of MPOS were mainly fructose and glucose while arabinose had prominent influence on prebiotic potentials. In the fermentation study, *B. animalis* TISTR 2195 was the preferred type based on its intracellular enzyme that could utilise the MPOS as a nutrition source. A higher amount of MPOS could generate greater fermented by-products. This is the first study to report sustainable use of the functional components derived from by-products of Thai mango processing in the form of pectic oligosaccharide resources that positively enhanced the growth of probiotics.

## Figures and Tables

**Table 1:** Chemical characteristics of mango peel pectin extracted from “chok anan” variety before hydrolysis and molecular weight and sugar content of MPOS.

Chemical Characteristic of “Chok Anan” Mango Peel Pectin (Initial MPP)	Pectin Yield (%)	Degree of Esterification (%)	Equivalent Weight (mg/mol)			Methoxyl Content (%)	
	15.06 ± 0.29	56.88 ± 0.78	1037.30 ± 4.96			4.00 ± 0.03	
Hydrolysis Time (h)	Enzyme Concentration (%*v/v*)	Molecular Weight (Da)	Monosaccharide Content (%*w/w*)				
			Glucose	Fructose	Galactose	Arabinose	Xylose
Initial MPP	-	-	6.28 ± 0.03	5.43 ± 0.08	0.75 ± 0.03	0.25 ± 0.02	Tr
6	0.1	<1000 (790)	14.08 ± 0.23 ^e^	18.79 ± 0.15 ^e^	2.07 ± 0.00 ^i^	1.07 ± 0.04 ^i^	Tr
	0.2	<1000 (759)	14.35 ± 0.18 ^e^	18.72 ± 0.14 ^e^	2.18 ± 0.00 ^h^	1.33 ± 0.04 ^h^	Tr
	0.3	<1000 (737)	14.37 ± 0.17 ^e^	18.99 ± 0.13 ^e^	2.34 ± 0.02 ^g^	1.47 ± 0.00 ^g^	Tr
12	0.1	<1000 (697)	15.61 ± 0.30 ^d^	20.47 ± 0.23 ^d^	2.76 ± 0.00 ^f^	1.68 ± 0.07 ^f^	Tr
	0.2	<1000 (693)	16.52 ± 0.00 ^c^	20.56 ± 0.40 ^d^	2.81 ± 0.03 ^e^	1.91 ± 0.00 ^e^	Tr
	0.3	<1000 (681)	17.93 ± 0.30 ^b^	21.43 ± 0.19 ^c^	2.88 ± 0.02 ^d^	2.13 ± 0.03 ^d^	Tr
24	0.1	<1000 (666)	19.01 ± 0.19 ^a^	22.49 ± 0.16 ^b^	3.05 ± 0.02 ^c^	2.31 ± 0.02 ^c^	Tr
	0.2	<1000 (660)	19.21 ± 0.48 ^a^	22.70 ± 0.23 ^b^	3.10 ± 0.01 ^b^	2.40 ± 0.01 ^b^	Tr
	0.3	<1000 (643)	19.52 ± 0.55 ^a^	24.41 ± 1.02 ^a^	3.35 ± 0.01 ^a^	3.02 ± 0.03 ^a^	Tr
Time (T)		-	*	*	*	*	n/a
Enzyme concentration (E)		-	*	*	*	*	n/a
T*E		-	*	*	*	*	n/a

The result of the molecular weight detected <1000 Da in all samples using ultrahydrogel linear 1 column. Numbers in the brackets are the M_z_ values reported from the built-in software. Average ± standard deviation with different subscription letters in each row (treatment) is significantly different (*p* < 0.05). Subscription (*) indicates significantly difference (*p* < 0.05) using two-way ANOVA with Duncan’s multiple range test. Monosaccharide concentrations were expressed as g/100 g dry weight. Tr = trace amount (<0.01% *w/w*). n/a = not available.

**Table 2:** ΔLA/ΔSCFA of each probiotic bacterial strain in the media supplemented with different carbon sources cultivated for 72 h of fermentation time.

Carbon Sources	*L. reuteri* DSM 17938	*B. animalis* TISTR 2195
Control	0.08 ± 0.00 ^Da^	0.05 ± 0.00 ^Da^
1% MPOS	0.01 ± 0.00 ^Fb^	0.24 ± 0.02 ^Ca^
2% MPOS	0.06 ± 0.00 ^Eb^	0.41 ± 0.00 ^Ba^
4% MPOS	0.62 ± 0.00 ^Ba^	0.002 ± 0.00 ^Eb^
2%MPP	0.13 ± 0.01 ^Ca^	0.03 ± 0.01 ^DEb^
2%Glucose	4.18 ± 0.04 ^Aa^	0.96 ± 0.05 ^Ab^

Average ± standard deviation with different capital letters in each column of each probiotic strain is significantly different (*p* < 0.05) and average ± standard deviation with different lowercase letters in each row is statistically significantly different (*p* < 0.05) between bacterial strains.

## Data Availability

Not applicable.

## Supplementary Materials

The following are available online at https://www.mdpi.com/2304-8158/10/3/627/s1, Table S1: Short chain fatty acid and lactic acid production of probiotic bacteria cultivated in medium added with various carbon sources at 24 to 72 h of fermentation time by *L. reuteri* DSM 17938, Table S2. Short chain fatty acid and lactic acid production of probiotic bacteria cultivated in medium added with vari-ous carbon sources at 24 to 72 h of fermentation time by *B. animalis* TISTR 2195, Table S3. Statistical analysis data using two-way ANOVA with Duncan’s multiple range test (*p* < 0.05) of optimisation condition of MPOS on glucose content in MPEP, Table S4. Statistical analysis data using two-way ANOVA with Duncan’s multiple range test (*p* < 0.05) of optimisation condition of MPOS on fructose content in MPEP, Table S5. Statistical analysis data using two-way ANOVA with Duncan’s multiple range test (*p* < 0.05) of optimisation condition of MPOS on galactose content in MPEP, Table S6. Statistical analysis data using two-way ANOVA with Duncan’s multiple range test (*p* < 0.05) of optimisation condition of MPOS on arabinose content in MPEP, Table S7. Statistical analysis data using two-way ANOVA with Duncan’s multiple range test (*p* < 0.05) of MPOS on prebiotic ac-tivity scores of *L. reuteri*, Table S8. Statistical analysis data using two-way ANOVA with Duncan’s multiple range test (*p* < 0.05) of MPOS on prebiotic ac-tivity scores of *B. animalis*.
