# Supplementary material for: Crude Pectic Oligosaccharide Recovery from Thai Chok Anan Mango Peel Using Pectinolytic Enzyme Hydrolysis
Source: Foods. 2021 Mar 16;10(3):627. doi: 10.3390/foods10030627 (PMC7999440; doi:10.3390/foods10030627)
Supplement: Supplementary file 1 [file foods-10-00627-s001.pdf]

Table S1. Short chain fatty acid and lactic acid production of probiotic bacteria cultivated in medium added with various carbon sources at 24 to 72 hours of fermentation time by *L. reuteri* DSM 17938.

Organic Acid (mM)	Fermentation Time (h)	Carbon Sources					
		**Control**	**1%MPOS**	**2%MPOS**	**4%MPOS**	**2%MPP**	**2%Glucose**
Acetic acid	24	7.78 ± 0.05 ^{Kb}	7.35 ± 0.05 ^{Kb}	7.62 ± 0.06 ^{Kc}	7.63 ± 0.37 ^{Kc}	10.70 ± 0.50 ^{Hc}	12.51 ± 0.10 ^{EFb}
	48	9.42 ± 0.01 ^{Ia}	7.69 ± 0.09 ^{Kb}	8.45 ± 0.07 ^{Jb}	18.27 ± 0.29 ^{Bb}	11.54 ± 0.07 ^{Gb}	12.65 ± 0.28 ^{Eb}
	72	9.43 ± 0.18 ^{Ia}	12.18 ± 0.13 ^{Da}	13.18 ± 0.07 ^{Fa}	22.68 ± 0.51 ^{Aa}	14.90 ± 0.50 ^{Ca}	18.35 ± 0.38 ^{Ba}
Propionic acid	24	2.53 ± 0.02 ^{Lc}	3.37 ± 0.02 ^{Kc}	3.70 ± 0.06 ^{Kc}	25.36 ± 0.32 ^{Ec}	16.52 ± 0.08 ^{Hc}	20.24 ± 0.31 ^{Fb}
	48	3.58 ± 0.08 ^{Kb}	4.56 ± 0.06 ^{Jb}	4.32 ± 0.04 ^{Jb}	29.23 ± 0.48 ^{Bb}	18.65 ± 0.24 ^{Gb}	20.66 ± 0.09 ^{Fb}
	72	5.04 ± 0.96 ^{Ia}	28.75 ± 0.26 ^{BCa}	28.54 ± 0.35 ^{Ca}	34.22 ± 0.49 ^{Aa}	26.20 ± 0.14 ^{Da}	25.61 ± 0.36 ^{Ea}
Isobutyric acid	24	1.73 ± 0.08 ^{Hc}	0.46 ± 0.02 ^{MNc}	0.70 ± 0.01 ^{Ic}	0.44 ±0.01 ^{MNc}	2.29 ± 0.14 ^{Fa}	0.62 ± 0.02 ^{JKc}
	48	2.01 ± 0.02 ^{Gb}	2.10 ± 0.02 ^{Lb}	2.84 ± 0.02 ^{Cb}	4.46 ± 0.21 ^{Aa}	0.33 ± 0.01 ^{Oc}	2.11 ± 0.04 ^{Gb}
	72	2.38 ± 0.02 ^{DFa}	4.29 ± 0.07 ^{Ja}	3.57 ± 0.14 ^{Ca}	0.60 ± 0.02 ^{JKb}	0.48 ± 0.01 ^{Mb}	2.49 ± 0.12 ^{Ea}
Butyric acid	24	0.43 ± 0.01 ^{Gc}	0.43 ± 0.02 ^{Gb}	0.44 ± 0.01 ^{Gb}	0.45 ± 0.01 ^{FGc}	0.43 ± 0.01 ^{Gc}	0.43 ± 0.02 ^{Gc}
	48	0.56 ± 0.03 ^{Db}	0.51 ± 0.01 ^{Ea}	0.45 ± 0.02 ^{FGb}	0.56 ± 0.01 ^{Db}	0.55 ± 0.01 ^{Db}	0.48 ± 0.02 ^{EFb}
	72	1.09 ± 0.04 ^{Ba}	0.54 ± 0.02 ^{Da}	0.47 ± 0.01 ^{EFa}	1.70 ± 0.02 ^{Aa}	0.71 ± 0.02 ^{Ca}	0.56 ± 0.01 ^{Da}
Isovaleric acid	24	0.29 ± 0.01 ^{Jc}	0.86 ± 0.06 ^{Gc}	0.68 ± 0.01 ^{Hc}	0.09 ± 0.00 ^{Kc}	0.29 ± 0.01 ^{Jc}	0.09 ± 0.00 ^{Kc}
	48	0.58 ± 0.03 ^{Ib}	1.41 ± 0.02 ^{Fb}	1.52 ± 0.03 ^{DEb}	1.51 ± 0.01 ^{Eb}	0.34 ± 0.02 ^{Jb}	3.03 ± 0.04 ^{Bb}
	72	0.67 ± 0.04 ^{Ha}	1.55 ± 0.01 ^{DEa}	2.10 ± 0.04 ^{Ca}	1.58 ± 0.02 ^{Da}	0.63 ± 0.02 ^{HIa}	3.54 ± 0.11 ^{Aa}
Valeric acid	24	0.31 ± 0.01 ^{Mc}	0.30 ± 0.01 ^{Mc}	0.23 ± 0.02 ^{Mc}	2.26 ± 0.02 ^{Jc}	2.60 ± 0.14 ^{Ic}	5.48 ± 0.02 ^{Hb}
	48	0.68 ± 0.02 ^{Lb}	1.49 ± 0.04 ^{FEb}	6.55 ± 0.12 ^{Kb}	5.81 ± 0.02 ^{EFb}	2.75 ± 0.15 ^{Ib}	5.68 ± 0.05 ^{FGb}
	72	0.76 ± 0.02 ^{La}	5.56 ± 0.06 ^{GHa}	8.43 ± 0.13 ^{Ja}	7.79 ± 0.14 ^{Ba}	5.92 ± 0.13 ^{Ea}	6.75 ± 0.16 ^{Ca}
Total SCFA	24	13.07	12.77	13.77	36.23	32.83	39.31
	48	16.83	17.76	41.49	59.84	34.16	44.61
	72	19.37	52.87	56.29	68.57	48.84	57.30
Lactic acid	24	9.70 ± 0.11 ^{Mb}	14.31 ± 0.03 ^{Jb}	23.28 ± 0.26 ^{Hc}	26.00 ± 0.22 ^{Fc}	13.10 ± 0.27 ^{Kc}	75.27 ± 0.54 ^{Cc}
	48	9.85 ± 0.03 ^{Mb}	14.52 ± 0.32 I^{Jab}	25.07 ± 0.33 ^{Gb}	37.99 ± 0.19 ^{Eb}	13.99 ± 0.33 ^{Jb}	121.50 ± 0.40 ^{Bb}
	72	10.32 ± 0.01 ^{La}	14.81 ± 0.19 ^{Ia}	25.61 ± 0.25 ^{Fa}	45.24 ± 0.23 ^{Da}	14.87 ± 0.12 ^{Ia}	150.49 ± 0.43 ^{Aa}
ΔL/ΔSCFA		0.08 ± 0.00 ^{D}	0.01 ± 0.00 ^{F}	0.06 ± 0.00 ^{E}	0.62 ± 0.00 ^{B}	0.13 ± 0.01 ^{C}	4.18 ± 0.04 ^{A}

Average ± standard deviation with different capital letters in each column of each organic acid are significantly different ($p < 0.05$) and average ± standard deviation with different lowercase letters is significantly different ($p < 0.05$) between short chain fatty acid values within the same carbon source of each organic acid.

Table S2. Short chain fatty acid and lactic acid production of probiotic bacteria cultivated in medium added with various carbon sources at 24 to 72 hours of fermentation time by *B. animalis* TISTR 2195.

Organic Acid (mM)	Fermentation Time (h)	Carbon Sources					
		**Control**	**0%MPOS**	**1%MPOS**	**2%MPOS**	**2%MPP**	**2%Glucose**
Acetic acid	24	1.42 ± 0.06 ^{Nc}	5.70 ± 0.09 ^{Lc}	6.58 ± 0.04 ^{Kc}	8.44 ± 0.04 ^{Ic}	8.03 ± 0.03 ^{Ic}	15.38 ±0.12 ^{Gb}
	48	1.98 ± 0.07 ^{Nb}	8.08 ± 0.01 ^{Jb}	12.60 ± 0.07 ^{Hb}	12.72 ± 0.07 ^{Hb}	17.62 ± 0.11 ^{Eb}	17.45 ± 0.04 ^{Da}
	72	3.39 ± 0.05 ^{Ma}	16.54 ± 0.08 ^{Fa}	25.17 ± 1.01 ^{Ba}	26.85 ± 0.32 ^{Aa}	18.42 ±0.25 ^{Ca}	17.69 ± 0.03 ^{Da}
Propionic acid	24	3.42 ± 0.01 ^{Lb}	9.30 ± 0.07 ^{Cc}	3.74 ± 0.02 ^{Kc}	4.63 ± 0.01 ^{Hc}	3.48 ± 0.02 ^{Lc}	4.04 ± 0.02 ^{Jc}
	48	3.43 ± 0.01 ^{Lb}	9.81 ± 0.07 ^{Bb}	4.85 ± 0.12 ^{Gb}	4.88 ± 0.02 ^{Gb}	4.07 ± 0.02 ^{IJb}	4.15 ± 0.02I ^{Jb}
	72	5.56 ± 0.19 ^{Ea}	12.81 ± 0.14 ^{Aa}	8.16 ±0.10 ^{Da}	5.26 ± 0.02 ^{Fa}	4.18 ± 0.01 ^{Ia}	4.84 ± 0.02 ^{Ga}
Isobutyric acid	24	1.24 ± 0.00 ^{Kc}	1.33 ± 0.02 ^{Jb}	0.88 ± 0.00 ^{Nc}	0.94 ± 0.01 ^{Ma}	1.12 ± 0.01 ^{Lc}	1.28 ± 0.02 ^{Kc}
	48	7.82 ± 0.10 ^{Bb}	1.60 ± 0.01 ^{Ha}	2.49 ± 0.02 ^{Fb}	1.40 ± 0.01 ^{Ib}	1.81 ± 0.01 ^{Gb}	1.59 ± 0.03 ^{Hb}
	72	9.47 ± 0.04 ^{Aa}	1.63 ± 0.02 ^{Ha}	3.71 ± 0.02 ^{Ca}	2.49 ± 0.02 ^{Fa}	2.67 ± 0.02 ^{Da}	2.58 ± 0.02 ^{Ea}
Butyric acid	24	0.50 ± 0.00 ^{Fc}	0.34 ± 0.02 ^{JKc}	0.32 ± 0.01 ^{Lc}	0.32 ± 0.01 ^{KLc}	0.35 ± 0.01 ^{Jc}	0.31 ± 0.01 ^{La}
	48	1.88 ± 0.01 ^{Bb}	0.43 ± 0.01 ^{Gb}	0.49 ± 0.02 ^{Fb}	0.37 ± 0.01 ^{Ib}	0.44 ± 0.01 ^{Gb}	0.39 ± 0.00 ^{Hb}
	72	2.19 ± 0.01 ^{Aa}	0.65 ± 0.01 ^{Da}	0.57 ± 0.02 ^{Ea}	0.57 ± 0.01 ^{Ea}	0.49 ± 0.01 ^{Fa}	1.03 ± 0.02 ^{Ca}
Isovaleric acid	24	0.14 ± 0.00 ^{Kc}	0.15 ± 0.00 ^{Kc}	0.18 ± 0.01 ^{Kc}	0.32 ± 0.01 ^{HIc}	0.24 ± 0.01 ^{Jc}	0.31 ± 0.01 ^{HIb}
	48	2.50 ± 0.03 ^{Bb}	0.41 ± 0.01 ^{EFb}	0.30 ± 0.01 ^{Ib}	0.43 ± 0.01 ^{Eb}	0.35 ± 0.02 ^{GHb}	0.37 ± 0.02 ^{FGa}
	72	4.98 ± 0.10 ^{Aa}	0.57 ± 0.02 ^{Ca}	0.42 ± 0.02 ^{Ea}	0.56 ± 0.01 ^{Ca}	0.48 ± 0.00 ^{Da}	0.38 ± 0.01 ^{EFGa}
Valeric acid	24	2.76 ± 0.02 ^{Hc}	4.86 ± 0.02 ^{Ec}	1.37 ± 0.04 ^{Jc}	0.33 ± 0.01 ^{NOb}	0.23 ± 0.01 ^{Pb}	0.87 ± 0.01 ^{Mb}
	48	3.72 ± 0.02 ^{Gb}	5.47 ± 0.16 ^{Db}	3.91 ± 0.01 ^{Fb}	0.34 ± 0.01 ^{NOb}	0.35 ± 0.01 ^{NOa}	0.93 ± 0.01 ^{KLa}
	72	6.71 ± 0.09 ^{Ba}	7.25 ± 0.29 ^{Aa}	5.90 ± 0.02 ^{Ca}	1.69 ± 0.01 ^{Ia}	0.39 ± 0.01 ^{Na}	1.01 ± 0.02 ^{Ka}
Total SCFA	24	9.48	21.68	13.07	14.97	13.45	22.19
	48	21.32	25.80	24.64	20.14	24.64	24.88
	72	32.30	39.45	43.93	69.15	26.63	27.53
Lactic acid	24	7.23 ± 0.02 ^{Kb}	14.51 ± 0.12 ^{Hc}	9.04 ± 0.08 ^{Ic}	1.48 ± 0.01 ^{Nb}	1.89 ± 0.09 ^{Mb}	19.75 ± 0.06 ^{Ec}
	48	7.40 ± 0.35 ^{Kb}	16.26 ± 0.09 ^{Gb}	20.16 ± 0.43 ^{Db}	1.54 ± 0.03 ^{Na}	2.12 ± 0.06L ^{Ma}	22.25 ± 0.16 ^{Bb}
	72	8.42 ± 0.07 ^{Ja}	18.84 ± 0.17 ^{Fa}	21.64 ± 0.07 ^{Ca}	1.58 ± 0.02 ^{Na}	2.27 ± 0.02 ^{La}	24.89 ± 0.23 ^{Aa}
ΔL/ΔSCFA		0.05 ± 0.00 ^{D}	0.24 ± 0.02 ^{C}	0.41 ± 0.00 ^{B}	0.002 ± 0.00 ^{E}	0.03 ± 0.01 ^{DE}	0.96 ± 0.05 ^{A}

Average ± standard deviation with different capital letters in each column of each organic acid are significantly different ($p < 0.05$) and average ± standard deviation with different lowercase letters is significantly different ($p < 0.05$) between short chain fatty acid values within the same carbon source of each organic acid.

Table S3. Statistical analysis data using two-way ANOVA with Duncan's multiple range test ($p < 0.05$) of optimisation condition of MPOS on glucose content in MPEP.

Tests of Between-Subjects Effects					
Dependent Variable: Glucose					
**Source**	**Type III Sum of Squares**	**df**	**Mean Square**	***F***	**Significance**
Corrected Model	120.321 ^{a}	8	15.040	158.589	0.000
Intercept	7560.120	1	7560.120	79716.957	0.000
Time	111.580	2	55.790	588.272	0.000
Enzyme	3.977	2	1.988	20.967	0.000
Time ×Enzyme	4.764	4	1.191	12.559	0.000
Error	1.707	18	0.095	-	-
Total	7682.148	27	-		-
Corrected Total	122.028	26	-	-	-

a. *R* Squared = .986 (Adjusted *R* Squared = .980).

Table S4. Statistical analysis data using two-way ANOVA with Duncan's multiple range test ($p < 0.05$) of optimisation condition of MPOS on fructose content in MPEP.

Tests of Between-Subjects Effects<br>Dependent Variable: Fructose					
**Source**	**Type III Sum of Squares**	**df**	**Mean Square**	***F***	**Significance**
Corrected Model	94.461 [a]	8	11.808	74.644	0.000
Intercept	11852.463	1	11852.463	74927.766	0.000
Time	85.992	2	42.996	271.808	0.000
Enzyme	5.895	2	2.947	18.632	0.000
Time × Enzyme	2.574	4	0.644	4.069	0.016
Error	2.847	18	0.158	-	-
Total	11949.771	27	-	-	-
Corrected Total	97.308	26	-	-	-

a. *R* Squared = .971 (Adjusted *R* Squared = .958).

Table S5. Statistical analysis data using two-way ANOVA with Duncan's multiple range test ($p < 0.05$) of optimisation condition of MPOS on galactose content in MPEP.

Tests of Between-Subjects Effects<br>Dependent Variable: Galactose					
**Source**	**Type III Sum of Squares**	**df**	**Mean Square**	***F***	**Significance**
Corrected Model	4.665 [a]	8	0.583	2156.788	0.000
Intercept	200.846	1	200.846	742856.110	0.000
Time	4.380	2	2.190	8100.918	0.000
Enzyme	0.248	2	0.124	457.904	0.000
Time × Enzyme	0.037	4	0.009	34.164	0.000
Error	0.005	18	0.000	-	-
Total	205.516	27	-	-	-
Corrected Total	4.670	26	-	-	-

a. *R* Squared = .999 (Adjusted *R* Squared = .998).

Table S6. Statistical analysis data using two-way ANOVA with Duncan's multiple range test ($p < 0.05$) of optimisation condition of MPOS on arabinose content in MPEP.

Tests of Between-Subjects Effects<br>Dependent Variable: Arabinose					
**Source**	**Type III Sum of Squares**	**df**	**Mean Square**	***F***	**Significance**
Corrected Model	8.909 [a]	8	1.114	1069.999	0.000
Intercept	99.994	1	99.994	96079.772	0.000
Time	7.466	2	3.733	3587.071	0.000
Enzyme	1.240	2	0.620	595.548	0.000
Time × Enzyme	0.203	4	0.051	48.689	0.000
Error	0.019	18	0.001	-	-
Total	108.922	27	-	-	-
Corrected Total	8.927	26	-	-	-

a. *R* Squared = .998 (Adjusted *R* Squared = .997).

Table S7. Statistical analysis data using two-way ANOVA with Duncan's multiple range test ($p < 0.05$) of MPOS on prebiotic activity scores of *L. reuteri*.

Tests of Between-Subjects Effects<br>Dependent Variable: Prebiotic Activity Scores of *L. reuteri*					
**Source**	**Type III Sum of Squares**	**df**	**Mean Square**	***F***	**Significance**
Corrected Model	104.049 [a]	8	13.006	572.117	0.000
Intercept	123.863	1	123.863	5448.532	0.000
Time	47.674	2	23.837	1048.554	0.000
Enzyme	22.284	2	11.142	490.120	0.000
Time × Enzyme	34.091	4	8.523	374.897	0.000
Error	0.409	18	0.023	-	-
Total	228.322	27	-	-	-
Corrected Total	104.458	26	-	-	-

a. *R* Squared = .996 (Adjusted *R* Squared = .994).

Table S8. Statistical analysis data using two-way ANOVA with Duncan's multiple range test ($p < 0.05$) of MPOS on prebiotic activity scores of *B. animalis*.

Tests of Between-Subjects Effects Dependent Variable: Prebiotic Activity Scores of *B. animalis*					
**Source**	**Type III Sum of Squares**	**df**	**Mean Square**	***F***	**Significance**
Corrected Model	118.344[a]	8	14.793	1128.917	0.000
Intercept	165.912	1	165.912	12661.461	0.000
Time	52.137	2	26.068	1989.385	0.000
Enzyme	20.399	2	10.200	778.369	0.000
Time × Enzyme	45.808	4	11.452	873.958	0.000
Error	0.236	18	0.013	-	-
Total	284.492	27	-	-	-
Corrected Total	118.580	26	-	-	-

a. R Squared = .998 (Adjusted R Squared = .997).
